# Wnt/β-Catenin Signaling in Oral Carcinogenesis

**DOI:** 10.3390/ijms21134682

**Published:** 2020-06-30

**Authors:** Montserrat Reyes, Tania Flores, Diego Betancur, Daniel Peña-Oyarzún, Vicente A. Torres

**Affiliations:** 1 Department of Pathology and Oral Medicine, Faculty of Dentistry, Universidad de Chile, Santiago 8380453, Chile; tania.flores@ufrontera.cl (T.F.); diego.betancur@ug.uchile.cl (D.B.); 2 Institute for Research in Dental Sciences, Faculty of Dentistry, Universidad de Chile, Santiago 8380453, Chile; dpenaoya@ug.uchile.cl; 3 Research Centre in Dental Science (CICO), Faculty of Dentistry, Universidad de La Frontera, Temuco 4780000, Chile; 4 Advanced Center for Chronic Diseases (ACCDiS), Universidad de Chile, Santiago 8380453, Chile

**Keywords:** oral cancer, dysplasia, endosome, β-catenin, destruction complex, Rab5

## Abstract

Oral carcinogenesis is a complex and multifactorial process that involves cumulative genetic and molecular alterations, leading to uncontrolled cell proliferation, impaired DNA repair and defective cell death. At the early stages, the onset of potentially malignant lesions in the oral mucosa, or oral dysplasia, is associated with higher rates of malignant progression towards carcinoma in situ and invasive carcinoma. Efforts have been made to get insights about signaling pathways that are deregulated in oral dysplasia, as these could be translated into novel markers and might represent promising therapeutic targets. In this context, recent evidence underscored the relevance of the Wnt/β-catenin signaling pathway in oral dysplasia, as this pathway is progressively “switched on” through the different grades of dysplasia (mild, moderate and severe dysplasia), with the consequent nuclear translocation of β-catenin and expression of target genes associated with the maintenance of representative traits of oral dysplasia, namely cell proliferation and viability. Intriguingly, recent studies provide an unanticipated connection between active β-catenin signaling and deregulated endosome trafficking in oral dysplasia, highlighting the relevance of endocytic components in oral carcinogenesis. This review summarizes evidence about the role of the Wnt/β-catenin signaling pathway and the underlying mechanisms that account for its aberrant activation in oral carcinogenesis.

## 1. Introduction

Oral cancer is a subtype of head and neck cancer, representing the sixth most common malignancy in the world [1,2]. Following diagnosis, 40–50% of patients have a five-year survival, and, hence, oral cancer embraces a main problem for global public health [3,4,5,6,7,8]. With the goal of improving patient survival, emphasis has been made on the early detection, diagnosis and treatment of potentially malignant lesions, in order to prevent their progression towards oral cancer. In this context, efforts are focused on unraveling signaling pathways that are deregulated in potentially malignant lesions and oral cancer, as these could be translated into novel therapeutic targets [9,10,11,12,13]. However, unlike the extensive information available for other epithelial cancers, little is known about the molecular mechanisms accounting for the progression of early lesions and oral cancer. Specifically, studies have reported upregulation of the Wnt/β-catenin signaling pathway in oral cancer and in potentially malignant oral lesions, although the activation extent of this pathway varies according to the stage of oral carcinogenesis [11,14,15,16]. Here, we will review the literature that demonstrate the aberrant activation of Wnt/β-catenin in oral carcinogenesis, by first describing the components of the canonical Wnt signaling pathway and its transcriptional role over genes involved in proliferation and cell survival. Then, we will summarize the evidence that show the involvement of the Wnt/β-catenin pathway in oral cancer and potentially malignant lesions. We will also discuss the role of increased Wnt ligand secretion in oral carcinogenesis. Finally, we will propose an endocytosis-dependent mechanism that would explain the upregulation of the Wnt/β-catenin pathway in oral dysplasia. Understanding the role of the Wnt/β-catenin signaling pathway in oral carcinogenesis, especially in oral dysplasia, will be essential to providing alternative therapeutic approaches to improve the outcomes of the patients.

## 2. Canonical Wnt Pathway

Wnt signaling pathways encompass both canonical and the different noncanonical pathways and are involved in a variety of biological functions, such as cell differentiation, migration and proliferation [17,18]. Unlike the noncanonical pathway, the canonical Wnt pathway has been extensively studied in the context of cancer cell biology, because it is altered in several malignancies, including colon, melanoma, breast, lung and oral cancers [19,20,21,22,23]. Specifically, in the last decade, a body of evidence has shown that this pathway is upregulated in head and neck malignancies, namely oral cancer and premalignant oral lesions, although the mechanisms accounting for such alterations remain poorly understood [16,20,24,25,26,27]. Particularly, it is intriguing that the alterations of this pathway in oral cancer are unlikely due to mutations in the components of this pathway, but rather, due to increased ligand production, as it will be detailed in the following sections of this review [11,15,28,29,30,31,32,33].

**Figure 1:**
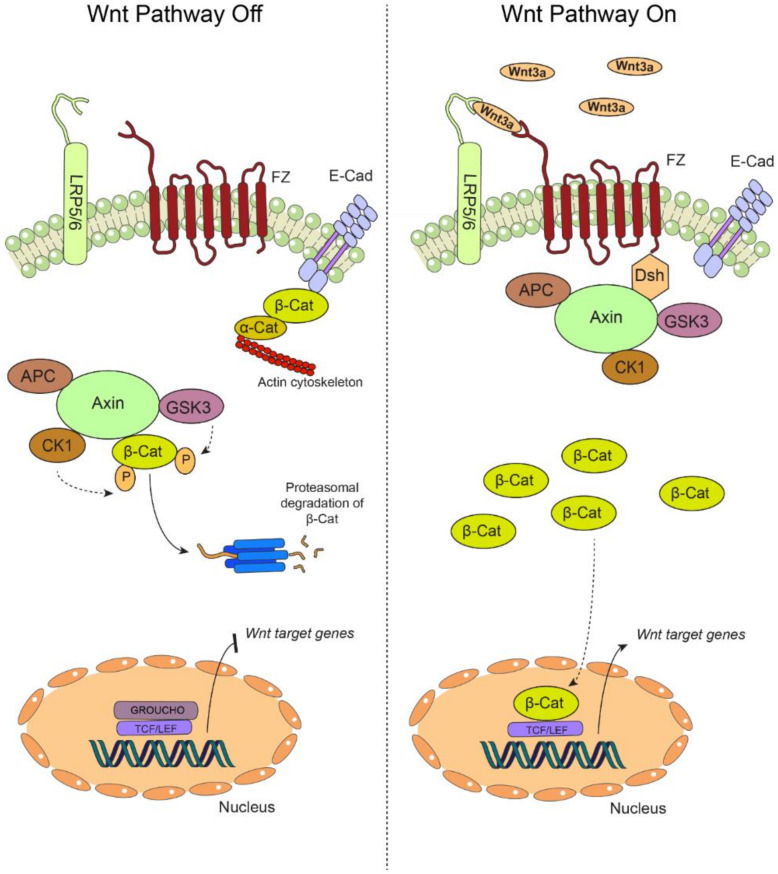
The canonical Wnt pathway. (**Left**) In the absence of extracellular Wnt ligands, the transmembrane receptors Frizzled (FZ) and the coreceptors LDL (low-density lipoprotein) receptor related protein 5/6 (LRP 5/6) are unable to associate at the plasma membrane, yielding an “off” state of the pathway. During this off state, β-catenin (β-Cat) is mainly found at cell-cell adhesion complexes, bridging together the intercellular adhesion molecule E-Cadherin (E-cad) and the actin cytoskeleton via interaction with α-catenin (α-Cat). In the cytoplasm, β-catenin is rapidly targeted for proteasomal degradation by the so-called “destruction complex”, which is composed by adenomatous polyposis coli (APC), axin, casein kinase 1 (CK1) and glycogen synthase kinase 3β (GSK3β). The targeting of β-catenin for degradation is based on sequential phosphorylation by CK1 and GSK3β. In these conditions, β-catenin cannot translocate to the nucleus, and the transcription of the target genes is repressed by GROUCHO, which is bound to the TCF/LEF promoters. (**Right**) Secreted Wnt ligands, such as Wnt3a, are recognized by both FZ and LRP5/6, switching “on” the pathway. The destruction complex is then recruited to the plasma membrane via interaction with the FZ receptor, allowing the cytoplasmic accumulation of β-catenin, which is now available for translocation to the nucleus, where it binds the TCF/LEF promoter by displacing GROUCHO, allowing the transcription of Wnt target genes. Particularly, in oral carcinogenesis, this pathway is “switched on” by the increased secretion of Wnt3a, stabilization of β-catenin and the expression of target genes such as *cyclin D1* and *survivin* (see main text for details).

The canonical Wnt pathway or Wnt/β-catenin signaling pathway is highly conserved in mammals and is activated by the binding of extracellular Wnt ligands to a membrane receptor in an autocrine/paracrine manner. Once activated, the canonical Wnt pathway induces the stabilization and nuclear translocation of β-catenin, which ultimately assists in the expression of genes involved in cell proliferation, viability, differentiation and migration [34,35]. In the absence of Wnt ligands, β-catenin is phosphorylated on residues Ser33/Ser37/Thr41 by a multiprotein complex, referred to as a “destruction complex”, which is formed by the enzyme glycogen synthase kinase 3β (GSK3β), casein kinase 1α (CK1α), the tumor-suppressor protein adenomatous polyposis coli (APC) and axin [36,37]. Phosphorylation of β-catenin leads to its degradation via proteasome and, subsequently, a decrease in cytoplasmic levels of β-catenin (Figure 1, left panel). As mentioned, activation of the Wnt/β-catenin pathway is initiated by the binding of the Wnt ligands to membrane receptors, namely the seven transmembrane receptors Frizzled (FZ) and the coreceptors LDL (low-density lipoprotein) receptor related protein 5/6 (LRP5/6) [36,38]. Then, this trimeric complex recruits the cytoplasmic protein Dishevelled (Dvl), which, in turn, sequesters the destruction complex, leading to the cytoplasmic stabilization of β-catenin [18,39,40]. Stabilized β-catenin in the cytoplasm is then able to translocate into the nucleus, forming a complex with T-cell factor/lymphoid enhancer factor (TCF/LEF) proteins to induce the transcription of genes involved in cell growth and proliferation, such as *c-myc*, *cyclin D1* and *survivin*, among others (Figure 1, right panel) [18,34,41]. Hence, as it might be anticipated, several of these target genes are upregulated in different cancers, including oral malignancies and potentially malignant lesions, as will be detailed in the upcoming sections.

## 3. Altered Wnt/β-Catenin Signaling in Oral Carcinogenesis

Aberrant activation of the Wnt/β-catenin signaling pathway is observed in different human cancers [17,40,42], and the most common alterations of this pathway are associated with the aberrant stabilization and nuclear translocation of β-catenin, as a consequence of inactivating mutations in APC or axin, as well as direct mutations in β-catenin [43,44,45,46,47,48], or the overexpression of Wnt ligands [11,49,50]. In this context, mutations in APC, axin and β-catenin are recurrent in colorectal cancer [21,51], whereas mutations in axin have been reported in esophagus squamous cell carcinoma and hepatocellular carcinoma [47,52,53]. In breast cancer, more than 50% of patients depict activated Wnt/β-catenin [23,54,55,56], which is associated with elevated incidences of metastasis [57]. On the other hand, the overexpression of Wnt ligands has been reported in different cancers—for instance, Wnt1 and Wnt5 in hepatocellular, colon or stomach cancers [58,59,60,61], Wnt5 in lung cancer [62,63,64] and Wnt3 in prostate tumors [65,66,67], as well as Wnt3 and Wnt5 in oral cancer [68,69,70,71].

Contrasting the extensive knowledge in other cancers, limited information is available about alterations in components of the Wnt pathway in oral carcinogenesis, and the mechanisms accounting for such alterations are just beginning to be elucidated. For instance, seemingly, and unlike other malignancies, no mutations in components of the Wnt/β-catenin pathway have been identified in oral cancer [28,30,31,33], and current explanations that support the deregulation of this pathway in oral cancer are based on the overproduction of Wnt ligands. This topic will be discussed later in this review. In the following section, we will review the literature that describes aberrant Wnt/β-catenin signaling in oral malignancies—first, by briefly introducing the process of oral carcinogenesis, then the upregulation of β-catenin in oral dysplasia, followed by its deregulation in oral cancer, and, finally, the role of Wnt inhibitors in oral carcinogenesis. In subsequent sections of this review, we will discuss current mechanisms proposed for β-catenin upregulation in oral cancer and oral dysplasia.

### 3.1. Oral Carcinogenesis

**Figure 2:**
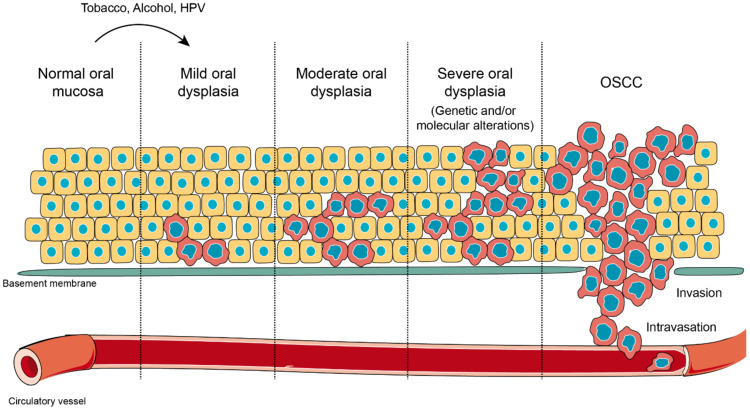
Oral carcinogenesis. Normal oral mucosa is a stratified layer of epithelial cells arranged over a basement membrane that separates epithelial cells from connective tissue and blood vessels. When oral mucosa is challenged with external stressors, such as tobacco, alcohol or human papilloma virus (HPV) infection, cells in the deepest layers undergo morphological alterations in shape and size. This novel state represents an adaptation response against a harmful stimulation, which is known as oral dysplasia. Oral dysplasia might be categorized as mild, moderate or severe, according with the extension of the lesion and the presence of molecular markers induced as result of the altered genetic expression. Oral dysplasia is considered the previous stage before oral squamous cell carcinoma (OSCC) and the strongest predictor of malignant transformation to cancer. During OSCC, massive phenotypic changes affect all epithelial layers, and it is extended over the tissue border, with ruptures of the basement membrane, in a process that allows the invasion of the connective tissue and incorporation into blood vessels (intravasation).

About 90% of oral cancers originate in the stratified nonkeratinized epithelium of the oral mucosa, which is the reason for its denomination as oral squamous cell carcinoma (OSCC), whose main risk factors include the consumption of tobacco and alcohol [2]. Oral carcinogenesis involves the accumulation of discrete and irreversible genetic alterations, as well as epigenetic changes that lead to altered expression/function in proteins, including p53, NOTCH1, EGFR, CDKN2A, STAT3, Cyclin D1, pRb and components of the Wnt/β-catenin pathway, among others [72,73,74,75,76,77,78]. The development of OSCC originates with the exposure towards a carcinogen that produces early genetic and molecular alterations in oral keratinocytes in all areas of tissue exposed to the carcinogen, which is followed by epithelial dysplasia in varying degrees of evolution, ending up with its malignant transformation to OSCC and metastasis (Figure 2) [6,79,80,81,82]. Initially, the molecular alterations in oral keratinocytes may not be expressed as clinical or histological lesions, increasing the risk of malignant transformation, which has been referred to as field cancerization, as proposed in 1953 by Slaughter et al. [83]. Field cancerization is clinically relevant in the prevention of groups of patients at high risk of developing oral cancer [84,85], because early genomic alterations, including microsatellite alterations, mutations in p53 and chromosomal instability, have been evidenced in otherwise histologically normal epithelium, adjacent to oral carcinomas [86,87]. The detection of genetically altered cells from clonal populations with increased growth and high proliferative rates indicate that lateral clonal extension is frequent in potentially malignant or invasive lesions. Therefore, early detection in asymptomatic stages is relevant, not only to permit an increase in survival rates, but also, to improve the quality of life as a consequence of using less aggressive and mutilating treatments, such as chemoprevention [88,89].

As mentioned, OSCC is preceded by lesions that are potentially malignant and histopathologically diagnosed as oral epithelial dysplasia, a stage that encompasses cellular and tissue alterations in the oral epithelium. Following the World Health Organization’s recommendations, two ways for classifying oral epithelial dysplasia have been proposed: one is the grading system for dysplasia, which has three categories: mild, moderate and severe dysplasia [7,90,91], whereas a more recent proposal is based on a binary categorization that considers only two states: low-grade dysplasia and high-grade dysplasia [92]. However, the first grading system remains the most widely used (Figure 2) [3,93,94]. Oral dysplasia is characterized by a modification in cell maturation within the epithelia, along with an increased proliferative activity, and, therefore, its diagnosis is the most important indicator of malignant potential [3,8,93,95]. Despite the knowledge that oral dysplasia is associated with high rates of progression towards invasive oral cancer, the mechanisms underlying the evolution of these lesions towards cancer are not fully understood. In this context, several studies have proposed the involvement of the Wnt/β-catenin pathway at the different stages of oral carcinogenesis, which expanded the knowledge about the molecular events involved in the progression from dysplasia towards oral cancer.

### 3.2. Wnt/β-Catenin Signaling in Oral Dysplasia

**Figure 3:**
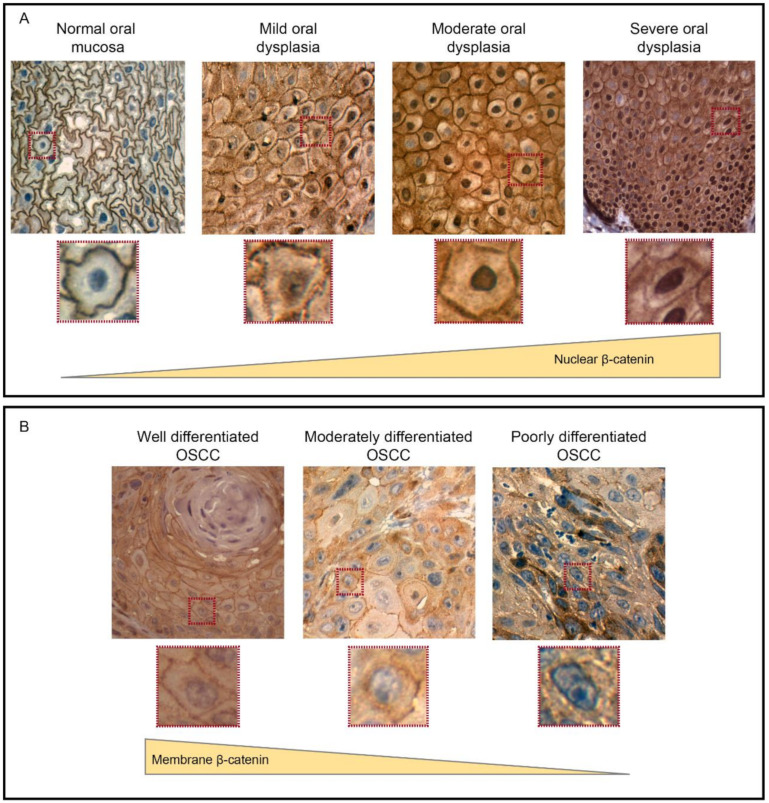
The expression and localization of β-catenin during oral carcinogenesis. (**A**) Immunohistochemistry of β-catenin in human oral samples, which revealed that β-catenin is mainly found at the plasma membrane of epithelial cells in normal oral mucosa samples. However, in oral dysplasia, β-catenin is mostly accumulated at the cytoplasm and nucleus of epithelial cells. Importantly, the nuclear detection of β-catenin is progressively increased according with the degree of dysplasia, with the strongest detection in severe and moderate oral dysplasia (zoomed images are shown in lower panels). (**B**) In OSCC, nuclear β-catenin levels are lower than oral dysplasia. On the other hand, plasma membrane-associated β-catenin decreases when shifting from well-differentiated to moderate and poorly differentiated OSCC (zoomed images are shown in the lower panels). This figure was modified with permission from “Increased nuclear β-catenin expression in oral potentially malignant lesions: A marker of epithelial dysplasia”, Reyes M. et al. 2015, Med Oral Patol Oral Cir Bucal.

Early studies by Sato et al. detected β-catenin at the nucleus in samples of oral dysplasia obtained from a murine model of carcinogenesis [16]. In accordance with these findings, our group found a high nuclear accumulation of β-catenin in biopsies obtained from patients diagnosed with oral dysplasia. In contrast, biopsies obtained from human donors with healthy oral mucosa depicted membranous localization only (Figure 3) [27], which is in accordance with the structural role that β-catenin plays in E-cadherin-mediated cell-cell junctions and epithelial cytoarchitecture, in the context that Wnt signaling is kept off. These data reconciled earlier studies, because they were consistent with reports showing that, in oral dysplasia, β-catenin is detected at the nucleus [15,26,96], while, at the oral carcinoma stage, this protein is mostly accumulated in the cytoplasm, with minimal detection in the nucleus [20,97]. Indeed, immunofluorescence and subcellular fractionation in vitro showed high levels of both total and non-phosphorylated (transcriptionally active) β-catenin in the nucleus of dysplastic oral keratinocytes when compared with nondysplastic oral keratinocytes and OSCC cells [11]. These observations are intriguing, as they are counterintuitive with those reported for other malignancies, where the progressive accumulation of nuclear β-catenin is observed throughout the whole process of carcinogenesis. However, interpretations should be carefully drawn from these observations, because little amounts of nuclear β-catenin might activate the transcription of target genes in OSCC, which is an issue that requires further exploration with appropriate methodologies, such as gene reporter assays and mRNA profiling. Additionally, at the cancer stage, routes other than Wnt/β-catenin, such as those triggered by the epidermal growth factor receptor via MAPK, might account not only for maintaining enhanced cell proliferation but, also, in the acquisition of the migration and invasion capabilities in OSCC cells [13,98,99]. Besides, we emphasize that the nuclear accumulation of β-catenin at the early stages of oral carcinogenesis is a phenomenon that might play a key role in the proliferation of dysplastic cells as a central event in oral dysplasia.

Unlike other malignancies, no mutations in components of the Wnt/β-catenin pathway, such as APC, axin or β-catenin itself, have been documented at the different stages of oral carcinogenesis [28,29,30,31], which suggests that mutations in these genes are unlikely to account for the nuclear accumulation of β-catenin in oral dysplasia and, hence, oral cancer. In this scenario, an interesting possibility to explain the aberrant activation of Wnt/β-catenin in oral dysplasia is the overproduction of Wnt ligands. In fact, immunohistochemical data showed the augmented expression of Wnt3a in oral leukoplakia [15]. In accordance with these observations, tissue coimmunofluorescence analysis showed a parallel and progressive increment in both Wnt3a expression and nuclear β-catenin when comparing normal mucosa with mild, moderate and severe dysplasia biopsies [11]. The same study showed that Wnt3a is the main Wnt ligand secreted by dysplastic oral keratinocytes and that, following secretion in cell cultures, this ligand is enough to promote the activation of Wnt/β-catenin in normal keratinocytes. In addition, the pharmacological inhibition of Wnt3a secretion by dysplastic oral keratinocytes interfered with the nuclear translocation of β-catenin and the induction of Wnt/β-catenin target genes, including *survivin* and *cyclin D1*, which raises the possibility of new therapeutic approaches for patients with oral dysplasia [11]. Altogether, during oral dysplasia, an exacerbation of Wnt/β-catenin activity might be attributed to an increased release of Wnt ligands. Moreover, as it will be discussed later, the epigenetic silencing of Wnt inhibitors, especially those molecules that interfere with ligand-receptor binding, might also explain the augmented activation of Wnt/β-catenin in oral dysplasia (see Section 3.4). Whether additional mechanisms contribute to β-catenin signaling in oral dysplasia remains unexplored—for instance, the PI3K-Akt pathway, which is known to promote the nuclear translocation of β-catenin in OSCC via the Akt-mediated phosphorylation of GSK3β [99,100,101]. On the other hand, studies in OSCC showed that EGFR overexpression [13,98] and subsequent activation promotes the nuclear translocation of β-catenin in oral cancer cells [102,103]; however, similar studies are not available in oral dysplasia, and further research is necessary to confirm a possible contribution of such pathways in β-catenin signaling during oral dysplasia. Collectively, events that lead to the upregulation of Wnt/β-catenin and consequent expression of target genes involved in cell viability and proliferation contribute to the acquisition of traits that are relevant to the early stages of oral carcinogenesis.

### 3.3. Wnt/β-Catenin Signaling in Oral Cancer

Unlike oral dysplasia, literature about the localization and function of β-catenin in oral cancer remains a matter of debate, although most studies suggest that β-catenin is mainly cytoplasmic and correlated with poor histological differentiation [104,105]. First, it should be remarked that, in normal oral mucosa, β-catenin is mainly found associated with E-cadherin, maintaining cell-to-cell interactions and preserving the architecture of the epithelium [104,105] (Figure 3). Conversely, in injured oral mucosa, non-membranous pools of β-catenin can be observed during the wound-healing process, which is associated with Axin2 positivity in proliferative Wnt-responsive re-epithelializing cells [106]. Besides, little is known about the role of Wnt/β-catenin in healthy oral mucosa, contrary to its well-described role in oral epithelial development [107].

The membranous expression of β-catenin exerts a restrictive effect on cell proliferation and migration, and, hence, the loss of β-catenin at this location is associated with increased cell motility and proliferation, correlating with the tumor grade and histological differentiation in OSSC [104,105]. In this context, focal β-catenin positivity has been observed in some studies [20,29,32,108,109]. However, unlike dysplasia, only 27% of samples with OSCC depicted nuclear β-catenin [11,27]. This phenomenon could be explained on the basis that the low nuclear localization of β-catenin is not necessarily translated into less transcriptional activity, because minimal amounts of β-catenin might be sufficient to allow transcriptional effects. To address this possibility, sophisticated approaches other than immunohistochemical analyses will be required. Conversely, most literature has been focused on demonstrating the tissue localization of this protein, rather than its transcriptional activity. Besides, it remains possible that the activation of Wnt/β-catenin is necessary only at the early stages of oral carcinogenesis, contributing to the increased rates of cell proliferation that are characteristic in oral dysplasia but following the onset of invasive OSCC; other signaling pathways become relevant to promote or sustain these effects.

Despite that several studies have explored the possibility of mutations in the components of the Wnt/β-catenin pathway in OSCC, no such mutations have been described so far [28,29,30,31]. Hence, it has been suggested that mutations in the components downstream of this pathway do not account for either cytoplasmic or sporadic nuclear localizations of β-catenin in OSCC cells, raising the possibility that Wnt ligands are involved. In this context, several Wnt ligands are overexpressed in oral cancer. For instance, high levels of Wnt3a are correlated with augmented nuclear β-catenin at the invasive front of OSCC [32]. Wnt5a, which is barely detected in normal oral mucosa, depicts a progressive increase at the oral dysplasia phase, reaching a maximal expression at the oral carcinoma stage [69,70]. On the other hand, Wnt7b has also been found overexpressed in OSCC, and it accounts for the activation of Wnt/β-catenin and tumor cell proliferation and invasion in vitro [110]. Finally, Wnt7a has been proposed as a promoter of OSCC progression by increasing the MMP-9 expression in a β-catenin-dependent manner [111]. Collectively, these evidences support the notion that Wnt ligands are relevant players in oral cancer progression.

In relation to Wnt/β-catenin target genes, reports have explored their expressions in oral cancer, with data showing abnormal expressions in a subset of those genes [112,113,114,115]. For example, high levels of cyclin D1, survivin and c-myc, which are classical Wnt/β-catenin targets, have been detected in OSCC samples when compared with control groups [76,116,117,118,119,120], indicating that Wnt/β-catenin target genes might be upregulated in oral cancer, contributing to cell migration and invasion.

Despite that most evidence has shown a low detection of membranous β-catenin in OSCC, and that augmented cytoplasmic β-catenin in invasive cancers is observed at the expense of decreased detection at the cell-cell junctions, this protein appears only sporadically located in the nucleus of OSCC. Hence, the assessment of functionality in this pathway becomes relevant, namely by exploring downstream transcriptions and their consequences in cell fates. This is due, in part, to the limited use of methodologies that are necessary to assess the status of Wnt/β-catenin-dependent events, such as gene expression. In this context, recent studies have explored the mechanistic aspects of β-catenin-dependent events in early lesions of the oral mucosa based on both in vitro models and in clinical samples (see the following sections).

### 3.4. Wnt Inhibitors in Oral Carcinogenesis

The Wnt/β-catenin signaling pathway is subject to regulation by Wnt antagonists, which include members of the Dickkopf family (Dkk), Wnt inhibitory factor 1 (WIF-1) and secreted frizzled-related proteins (SFRPs). Thus, a balanced function between the Wnt ligands and their inhibitors contributes to cell proliferation in normal tissues [37,121]. In different cancers, decreased expressions of these antagonists have been shown via epigenetic transcriptional silencing [35,122,123,124,125], and, in oral carcinogenesis, it has been reported that the silencing of these inhibitors (e.g., via DNA methylation) leads to cytoplasmic accumulation and the nuclear translocation of β-catenin with the concomitant activation of target genes such as *c-myc* and *cyclin D1* [71,108,126]. These events become noticeable when compared with healthy oral mucosa, where epigenetic silencing of these inhibitors is not frequent, β-catenin depicts a membranous expression and Wnt ligands are poorly expressed [71,108,126], despite the highly proliferative nature of oral mucosa.

In OSCC, members of the SFRP gene family, which prevent the binding of Wnt to frizzled receptors, are epigenetically downregulated via hypermethylation in their promoters [71]. Additionally, the role of SFRP2 in OSCC has been investigated by using in vivo and in vitro models, observing that the methylation of the SFRP2 promoter occurred more frequently in the tumor region than in the nontumor adjacent tissue and that the mRNA encoding for SFRP2 was significantly inhibited in OSCC biopsies [126]. In the same line, it has been suggested that changes in β-catenin localization are likely due to epigenetic modifications that affect the expression of SFRP and WIF-1 in OSCC [108], raising the possibility that methylation levels serve as possible prognostic approaches in OSCC [127]. In order to have a better understanding of the relevance of Wnt antagonists in oral carcinogenesis, new studies will be required. Likewise, the roles that these antagonists might play in premalignant lesions remain unexplored yet.

## 4. Mechanisms Involved in the Aberrant Activation of β-Catenin in Oral Cancer

### 4.1. Endosomal Trafficking and Wnt/β-Catenin Signaling

For years, the endocytic trafficking was considered as a mechanism of downregulation in signaling pathways, based on the fact that the internalization of the cell surface receptors and their subsequent transport from early endosomes to late endosomes and lysosomes leads to their degradation [128]. However, endocytosis plays a key role in the activation of signaling pathways, including the canonical Wnt pathway, since the internalization of the trimeric complex, formed by the Wnt ligand, Frizzled and LRP6, along with the β-catenin destruction complex, in endosomal compartments is necessary for the activation of the Wnt/β-catenin pathway [129].

Specifically, the binding of Wnt ligands causes phosphorylation of the cytoplasmic tail of LRP5/6 by GSK3β and CK1α. This phosphorylation enables the binding of the remaining proteins of the destruction complex at the plasma membrane, forming a structure known as “LRP6 signalosome”, which contains aggregates of Frizzled, phosphorylated LRP5/6, Dvl, axin, APC and GSK3β and whose complex is internalized in endosomal structures [129,130,131,132,133]. The inactivation of GSK3β has been considered as a key step in Wnt/β-catenin signaling, which begins with its binding to the LPR5/6 cytoplasmic tail and continues after its sequestration in early endosomes and multivesicular bodies. In this way, β-catenin cannot be phosphorylated by GSK3β, thus allowing its stabilization in the cytoplasm and subsequent translocation to the nucleus, where it activates the transcription of the target genes [134]. Although the mechanism of sequestration of the β-catenin destruction complex has been described, the role that components of the endocytic machinery play in this phenomenon has not been fully explored. In this scenario, recent studies have shown that endocytic proteins are relevant for Wnt/β-catenin signaling, thus providing insights into the mechanisms involved in β-catenin upregulation in oral cancer. In the following sections, we will discuss novel connections between endocytic trafficking and Wnt/β-catenin signaling in oral cancer by, firstly, introducing the role of endocytic trafficking in cancer and, then, its relevance in oral carcinogenesis via modulation of the Wnt/β-catenin pathway.

### 4.2. Deregulated Endocytosis in Cancer

A body of evidence accumulated in the past two decades supported the notion that uncontrolled endocytic trafficking is a recurrent phenomenon in malignancy, which is due, in part, to the fact that endosome trafficking controls a plethora of cellular processes [135,136]. In this context, it has been demonstrated that endocytic trafficking impacts at different aspects of the cell function, namely the availability of cell surface molecules that sustain cell signaling and cell interactions with the surrounding environment and by providing signaling platforms at the so-called signaling endosomes [129,137]. On the one hand, the endocytosis of cell adhesion molecules, such as E-cadherin, contribute to the epithelial-to-mesenchymal transition during tumor progression, whereas endocytic trafficking of cell-extracellular adhesion molecules, specifically integrins, along with the turnover of integrin-based focal adhesions, represent key events to sustain tumor cell migration, invasion and metastasis [137]. On the other hand, compartmentalization at the so-called “signaling endosomes” provides signaling platforms that orchestrate the activation of molecules involved in tumor cell proliferation, migration and metastasis, such as Rac1, MAPK and tyrosine kinases [129]. However, another layer of complexity is added by the fact that endosomes are also known to “switch off” negative regulators of signaling pathways, such as the Wnt/β-catenin pathway, for which sequestration of the destruction complex within multivesicular bodies allows β-catenin stabilization, nuclear translocation and the transcription of target genes [129,134]. Consequently, increasing studies have reported a deregulation of endocytic proteins in cancer, such as small GTPases, tethering molecules, effectors and adaptor proteins [135,137]. This is particularly observed for small GTPases of the Rab family of proteins, because they are master regulators of endosome trafficking and dynamics [138]. Specifically, Rab proteins are known to control cell adhesion, viability, anchorage independency, tumor cell migration, invasion and metastasis by a plethora of mechanisms, including enhanced integrin traffic [139,140,141,142], focal adhesion disassembly [143,144,145], Rho-GTPase balance [146,147,148] and mitosis [149], among many other events.

In oral cancer, alterations in endosomal proteins and regulators of intracellular trafficking have been reported, and these include the overexpression of Rab proteins [150,151], caveolin-1 [152] and the large GTPase GBP1 [153], whereas epigenetic silencing of the endocytic recycling regulator, Rab25, is associated with lymph node metastasis in oral and oropharyngeal squamous cell carcinomas [154,155]. Alternatively, the amplification of chromosomal regions encompassing genes that encode for Rab5, Rab7 and Rab11 has been associated with the upregulation of these GTPases in clinical samples of metastatic OSCC, and their expressions are associated with increased tumor cell migration and poor patient survival [151]. In another study, an immunohistochemical analysis showed an overexpression of Rab5 in 50% of cases of OSCC [156].

**Figure 4:**
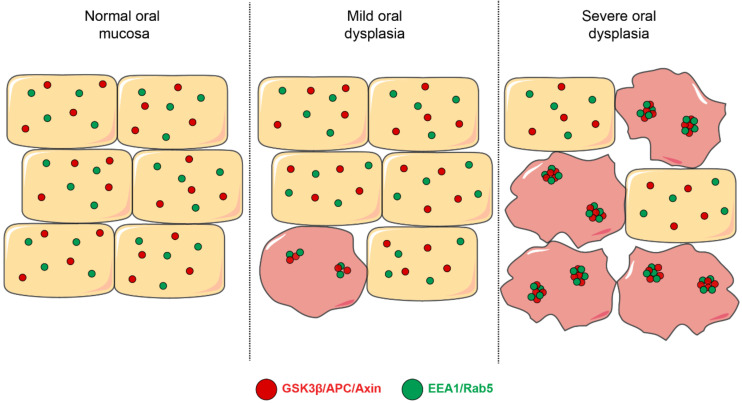
Early endosomes and their colocalization with components of the β-catenin destruction complex. This figure depicts a model that summarizes different aspects related to the mechanisms involved in Wnt/β-catenin signaling in oral dysplasia. First, early endosomes (shown in green, EEA1- and Rab5-positive) are progressively enlarged throughout the different stages of oral dysplasia in a manner that their co-localization with the components of the β-catenin destruction complex is also augmented during the progression of oral dysplasia. Specifically, the proteins APC, axin and GSK3β have been shown to increasingly colocalize with EEA1- and Rab5-positive early endosomes.

Based on these observations, it is relevant to explore possible consequences derived from Rab upregulation in OSCC, especially traits associated with malignancy. Intriguingly, whether these GTPases are altered at the early stages of oral mucosal carcinogenesis remains largely unknown. In this context, our recent studies indicate that a subset of Rab-GTPases are upregulated in oral dysplasia [14]. Specifically, the activity of Rab5, but not Rab7 or Rab11, is increased in dysplastic oral keratinocytes when compared with nondysplastic oral keratinocytes. In accordance with this, the progressive enlargement of early endosomes is detected through the different grades of oral dysplasia, as shown in biopsies of mild, moderate and severe dysplasia (Figure 4) [14]. Consequences of such upregulation are yet to be fully understood, but augmented Rab5 activity in oral dysplasia is known to be associated with the aberrant localization of β-catenin to the nucleus of dysplastic oral keratinocytes due to the augmented sequestration of the β-catenin destruction complex in endosomes (Figure 4), as it will be described in the following section.

### 4.3. Endosomal Sequestration of the Destruction Complex in Oral Dysplasia

**Figure 5:**
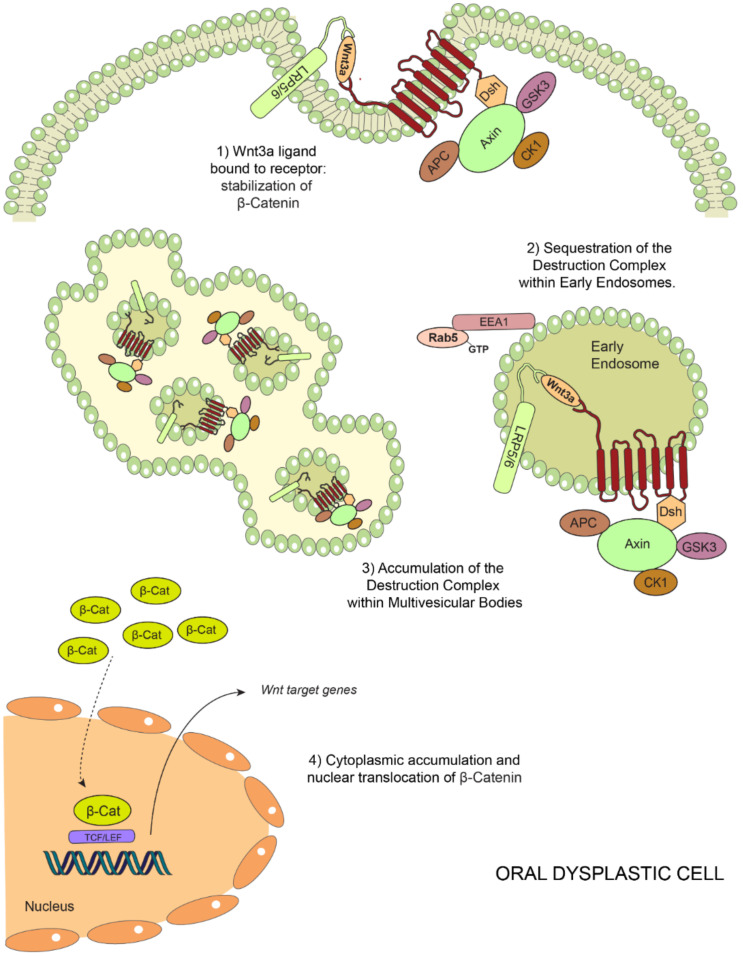
Model of endosomal sequestration of the destruction complex in oral dysplasia. Binding of Wnt3a to Frizzled (FZ) and LRP5/6 leads to the recruitment of the destruction complex to the FZ-LRP5/6 receptor complex at the plasma membrane. This is followed by a decreased proteasomal degradation of β-catenin (**1**). Ligand binding and subsequent posttranslational modifications (not described in this scheme) lead to endocytosis of this supramolecular complex, also known as the “Wnt signalosome complex”, and subsequent trafficking en route to early endosomes and multivesicular bodies in a Rab5-dependent manner (**2**). In oral dysplasia, high Wnt3a levels and increased Rab5 activity lead to the enhanced sequestration of the destruction complex within EEA1-positive early endosomes. Consequently, higher levels of components of the destruction complex are detected in multivesicular bodies (**3**). These events ultimately lead to a more robust stabilization of β-catenin in the cytoplasm and a consequent nuclear translocation in order to bind TCF/LEF factors, activating the transcription of the target genes (**4**).

Despite that the nuclear accumulation of β-catenin is recognized as a recurrent event in oral dysplasia, mechanisms accounting for such phenomenon have remained elusive [11,15,16,27]. Recently, a link was established between this phenomenon and components of the endosomal machinery in a model that proposes that augmented Rab5 activity accounts for early endosome enlargement in dysplastic oral keratinocytes [14]. This event is followed by the increased sequestration of components of the β-catenin destruction complex, including APC, axin and GSK3β, within EEA1- and Rab5-positive compartments (Figure 4). Although this study did not explore the fate of the β-catenin destruction complex, data suggested that a subset of proteins forming this complex are targeted en route to the endo-lysosomal degradative pathway in dysplastic oral keratinocytes, since both proteins GSK3β and axin appeared enriched in fractions that were resistant against the proteinase K treatment, suggesting their accumulation in multivesicular bodies [14] (see proposed model, Figure 5). Confirmation of these possibilities will require further assessments by alternative approaches, including colocalization analyses of components of the destruction complex and late endosome/lysosome markers, as well as electron microscopy for ultrastructural assessments, as previously described [134]. Alternatively, causal-effect studies in cell culture models would permit evaluating whether the inhibition of the endo-lysosomal system is followed by a diminished downregulation of the destruction complex, hence preventing the aberrant stabilization of β-catenin in dysplastic oral keratinocytes.

Although further research is still required, and that more mechanistic insights should be drawn by in vitro approaches, it must be emphasized that current evidence showing the endosomal sequestration of components of the β-catenin destruction complex in patient biopsies represents a landmark finding to understand the “deregulation” of this pathway during oral carcinogenesis [11,14]. Intriguingly, whether this mechanism remains upregulated at late stages of carcinogenesis, namely carcinoma in situ and frank invasive OSCC, is an issue that needs to be explored in order to identify potential markers for predicting oral cancer progression.

## 5. Therapeutic Approaches Based on Targeting Wnt Secretion

As discussed, the Wnt/β-catenin pathway appears to be relevant in several malignancies, and, hence, therapies aiming to target Wnt signaling represent attractive therapeutic approaches. For instance, recombinant proteins that minimize Wnt-Frizzled interactions or Wnt inhibition have been proposed and thought to impact the outcomes of cancers with deregulated Wnt ligand secretions [157,158,159,160]. In oral cancer, little has been explored about possible Wnt-based therapies, which is mainly due to the lack of understanding about the mechanisms involved in Wnt secretion, cellular targets and downstream signaling. As previously explained, the ligand-mediated activation of Wnt/β-catenin is the best-known mechanism associated with β-catenin stabilization and nuclear translocation in oral cancer cells and dysplastic keratinocytes, and, hence, the remaining paragraphs will be dedicated to discussing some possibilities in this scenario.

Following their translation at the endoplasmic reticulum, Wnt ligands are posttranslationally modified by palmitoylation and glycosylation [34,38,161]. Palmitoylation is achieved by the enzyme o-acyl-transferase porcupine (PORCN) in a step that is essential for Wnt ligand secretion [161,162,163]. With this information, studies have shown that the inhibition of PORCN with the compound Wnt-C59 delays tumor growth, as observed in a model of breast cancer in transgenic mice, further indicating that the use of such inhibitors is safe and feasible in preclinical models [49]. Likewise, Cheng et al. reported that Wnt-C59 suppresses the growth of tumors derived from nasopharyngeal carcinoma in a murine model [50]. In the same line, it has been shown that compound LGK974, which is a potent inhibitor of Wnt signaling, is highly effective in decreasing tumor growth in mice [164]. Collectively, these evidences indicate that the inhibition of Wnt secretion is efficient in decreasing tumor growth when facing the aberrant activation of the Wnt pathway.

Conversely to these descriptions, which have been focused on the cancer stage itself, no study is available about a potential use of these pharmacological inhibitors at earlier stages of oral carcinogenesis, whose targeting should be significant to preclude tumor progression. To this end, a more comprehensive understanding of the mechanisms involved in Wnt activation during oral carcinogenesis will be required to rationally propose effective therapies in oral cancer.

## 6. Conclusions and Perspectives

Consequences of uncontrolled Wnt/β-catenin signaling in oral cancer are becoming elucidated, with special emphasis on the early events of oral carcinogenesis. Particularly, an increase in the secretion of Wnt ligands has a fundamental role in the progression of oral cancer through its effect on the processes of tumorigenesis and metastasis. In this context, therapeutic approaches aiming to use Wnt inhibitors appear advantageous for cancers associated with the overexpression of Wnt ligands, since the evidence supports their safety and feasibility in preclinical models, with a powerful inhibitory effect on Wnt signaling. This is expected to be impactful, not only by reducing the growth of tumors caused by the aberrant activation of this pathway, but also, by bringing promising therapies of chemoprevention in order to manage early malignant lesions and prevent their progression towards cancer.

**Figure 6:**
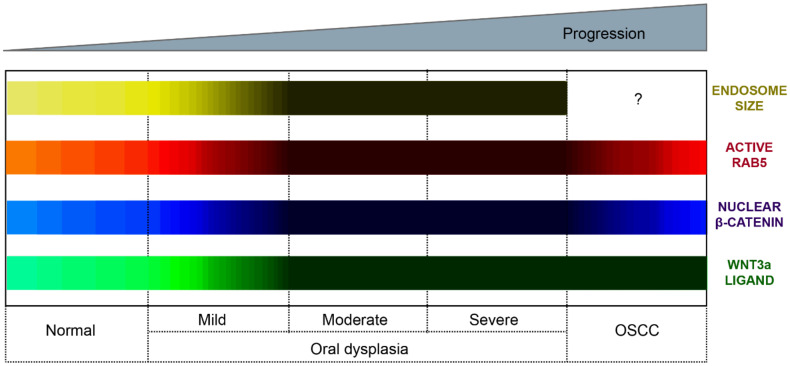
Subcellular and molecular events in oral carcinogenesis. This scheme provides a summary of subcellular and molecular changes observed during oral carcinogenesis. These changes include the Wnt3a expression, nuclear detection of β-catenin, endosome enlargement and the activation status of Rab5 GTPase. We propose a model whereby oral carcinogenesis is associated with the progressive expression of Wnt3a and the consequent stabilization and nuclear translocation of β-catenin. These events are accompanied by the continuous activation of Rab5-GTPase, endosome enlargement and the increased sequestration of the destruction complex within endosomes (see main text for details). Color codes represent the Wnt3a expression (green bar), nuclear β-catenin detection (blue bar), Rab5 activity (red bar) and early endosome size (yellow bar). The lighter the color, the lower the expression/detection/activity/size. The darker the color, the higher the expression/detection/activity/size for each case. The Wnt3a representation summarizes evidence obtained from immunohistochemical analyses in clinical samples and in vitro measurements of ligand secretions in cell culture models. The nuclear β-catenin representation summarizes evidence obtained in both clinical samples and cell culture models. The nuclear detection progressively increases from normal oral mucosa through the different stages of oral dysplasia (mild, moderate and severe); however, the nuclear detection of this protein decreases in OSCC. The Rab5 activity (Rab5-GTP levels) has been measured in cell culture models and is shown substantially increased in dysplastic oral keratinocytes (models of moderate/severe dysplasia) in comparison with nondysplastic oral keratinocytes and OSCC cells. The endosomal size (early endosomes) has been measured by tissue immunofluorescence in clinical samples of normal mucosa and mild, moderate and severe dysplasia, as well as in cell culture models, using different markers of early endosomes (the interrogation symbol indicates that this has not been explored in OSCC).

Recent studies indicate the involvement of endocytic proteins in promoting Wnt/β-catenin signaling during oral carcinogenesis, an effect namely attributed to their effects in the endosomal sequestration of the β-catenin destruction complex. Although these mechanisms are not completely understood, current models propose that augmented Rab5 activity accounts for early endosome enlargement, followed by increased sequestration of the components of the β-catenin destruction complex and the nuclear localization of β-catenin, which represents a landmark finding to understand the “deregulation” of this pathway during oral carcinogenesis (Figure 6). Further research is needed to have a comprehensive understanding of this mechanism and whether it remains upregulated at the late stages of oral carcinogenesis. This will also permit the identification and validation of possible markers to predict the progression of oral cancer and to propose potential, new and effective therapeutic objectives that favor both the quality of life and the survival of patients in the process of developing carcinoma in situ and frank invasive OSCC.
